# Sex, gender and gender identity: a re-evaluation of the evidence

**DOI:** 10.1192/bjb.2020.73

**Published:** 2021-10

**Authors:** Lucy Griffin, Katie Clyde, Richard Byng, Susan Bewley

**Affiliations:** 1 Priory Hospital Bristol, Bristol, UK; 2 Southern Health NHS Foundation Trust, Hampshire, UK; 3 Faculty of Health, University of Plymouth, UK; 4 Department of Women & Children's Health, King's College London, UK

**Keywords:** Sexual and gender identity disorders, ethics, comorbidity, phenomenology, consent and capacity

## Abstract

In the past decade there has been a rapid increase in gender diversity, particularly in children and young people, with referrals to specialist gender clinics rising. In this article, the evolving terminology around transgender health is considered and the role of psychiatry is explored now that this condition is no longer classified as a mental illness. The concept of conversion therapy with reference to alternative gender identities is examined critically and with reference to psychiatry's historical relationship with conversion therapy for homosexuality. The authors consider the uncertainties that clinicians face when dealing with something that is no longer a disorder nor a mental condition and yet for which medical interventions are frequently sought and in which mental health comorbidities are common.

In 2018 the Royal College of Psychiatrists (RCPsych) issued a position statement to promote good care when dealing with transgender and gender-diverse people that relates to ‘conversion therapy’.^1^ In this article we reappraise the phenomenology of gender identity, contrast ‘treatments’ for homosexuality with those for gender non-conformity, analyse the relationship between gender dysphoria and mental disorders with particular reference to the younger cohort of transgender patients, and ask how psychiatrists can address distress related to gender while upholding the central tenet of ‘first do no harm’.

## Homosexuality and conversion therapy

Male homosexuality was outlawed in the UK in 1865 until the Sexual Offences Act 1967 decriminalised sexual acts between men. During that time, homosexuality was shameful, stigmatised and conceptualised as a mental disorder. Psychiatry was instrumental in its treatment, which continued even after the legal change.^2^

Attempts to ‘cure’ same-sex desire included psychotherapy, hormone treatment and various behavioural interventions. These interventions are now considered ‘conversion’ or ‘reparative’ therapy.^3^ One high-profile failure for such ‘treatments’ was Alan Turing. After being found guilty of gross indecency in 1951, he was prescribed oestrogen, which rendered him impotent and caused gynaecomastia. He died by suicide in 1954.^4^

Conversion therapies lost popularity as evidence emerged of their ineffectiveness,^5^ coupled with more tolerant social attitudes. Homosexuality was removed from the World Health Organization (WHO) ICD-10 classification in 1992. In 2014, the RCPsych published a position statement explicitly rejecting conversion therapy and supporting a ban.^6^ Same-sex orientation is regarded as a normal, acceptable variation of human sexuality.

Enshrined in the Equality Act 2010, lesbians and gay men in the UK now enjoy the same civil rights as heterosexuals in terms of healthcare, marriage and raising of children, and equal employment. Although they enjoy equal status and increased visibility in most Western societies, there remain countries and cultures where same-sex practice is taboo or criminal, and where people still seek treatment.

## Beyond sexual orientation

In recent years, increasing links have been forged between lesbian and gay communities and those representing other gender identities. Stonewall describes ‘any person whose gender expression does not conform to conventional ideas of male or female’ as falling under the umbrella term ‘trans’.^7^

Definitions have evolved beyond those included in the 1992 ICD-10 under ‘gender identity disorders’, with which psychiatrists might be familiar.^8^ Transsexualism was widely understood to mean ‘a desire to live and be accepted as a member of the opposite sex, and an accompanied discomfort of one's anatomic sex’.^8^ Underlying mechanisms are poorly understood, although there are similarities and overlaps with both body dysmorphia and body integrity identity disorder.^9,10^ Sufferers might embark on social and medical intervention to help them ‘pass’ as the opposite sex. Historically, a diagnosis of gender dysphoria would have been required for doctors to intervene in this group.^11^

**Fig. 1:**
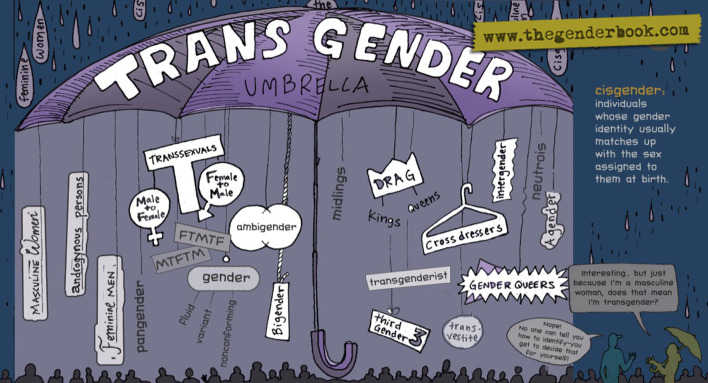
A page from *The Gender Book*^12^ (reproduced with permission of www.thegenderbook.com).

Transgender, however, has become a much broader category (Fig. 1). New terminology reflects a conceptual shift from clinical disorder to personal identity.^12^ Crucially, gender dysphoria is no longer integral to the condition. The World Health Organization has renamed ‘gender identity disorder’ as ‘gender incongruence’ and reclassified it as a ‘condition related to sexual health’ rather than retaining it in the chapter pertaining to ‘mental and behavioural disorders’,^13^ a somewhat discrepant placement, reflecting a political rather than scientific decision-making process.

By contrast, DSM-5 has removed ‘gender identity disorder’, renaming it ‘gender dysphoria’. It is possible to meet the criteria for a diagnosis of gender dysphoria within DSM-5 without experiencing body dysphoria relating to primary or secondary sexual characteristics,^14^ and the American Psychiatric Association emphasises that ‘not all transgender people suffer from gender dysphoria’.^15^

The following is from the 2018 ICD-11:^16^

‘Gender incongruence of childhood is characterized by a marked incongruence between an individual's experienced/expressed gender and the assigned sex in prepubertal children. It includes a strong desire to be a different gender than the assigned sex; a strong dislike on the child's part of his or her sexual anatomy or anticipated secondary sex characteristics and/or a strong desire for the primary and/or anticipated secondary sex characteristics that match the experienced gender; and make-believe or fantasy play, toys, games or activities and playmates that are typical of the experienced gender rather than the assigned sex.’

Definitions are inadequate in explaining how anyone experiences the gender of the opposite sex. Without further explanation of ‘toys, games or activities’ that are typical of each sex, this is left to parents, teachers and doctors to determine. The inference might be that gender-congruent behaviours have some objective existence and not fulfilling them might indicate a ‘trans’ identity. Children who do not conform to social norms and expectations come to dislike their sexual characteristics: that embodiment of their gender dissonance.

There is a lack of consensus demonstrated as to the exact nature of the condition. Questions remain for psychiatrists regarding whether gender dysphoria is a normal variation of gender expression, a social construct, a medical disease or a mental illness. If merely a natural variation, it becomes difficult to identify the purpose of or justification for medical intervention.

## Conversion therapy relating to gender

The RCPsych gives a description within the position statement of ‘treatments for transgender people that aim to suppress or divert their gender identity – i.e. to make them exclusively identify with the sex assigned to them at birth’.^1^ Conversion therapy is described as ‘any approach that aims to persuade trans people to accept their sex assigned at birth’. It goes on to include ‘placing barriers [to] medical transition’. Unfortunately, the statement does not define ‘approach’ beyond alluding to psychoanalytic or behavioural talking therapies. Thus, conversion therapy for transgender people appears conflated with that for homosexuality. Furthermore, there is little evidence that it is taking place in the UK.^17^ Historically, a diagnosis of gender dysphoria was required before medical intervention;^10^ this is a part of standard gatekeeping that is now being criticised as a ‘barrier’ instead of regular safe medical practice.^2^ Now, a self-declaration of being ‘trans’ appears to be indication enough for a patient to expect their doctor provide a range of complex medical treatments, with no evidence of dysphoria being required.^18^

The position statement^1^ could also be read as suggesting that full medical transition is an ultimate goal in gender-diverse patients, rather than considering a range of possible goals, which might include limited interventions or reconciliation with one's own (sexed) body. With regard to conversion therapy in children, the statement does not refer to desistance; evidence suggests that the majority of children left alone reconcile their identity with their biological sex; the feelings of 60–80% of children with a formal diagnosis of gender dysphoria remit during adolescence.^19–21^

## Definitions of sex, gender and gender identity

Gender theorists propose that all people must have a gender identity; it is not waivable. For those people whose internal identity aligns with their sex, the word cisgender and ‘cis’ terminology are used. Those whose identity is wholly that of the opposite sex are described as transgender or ‘trans’. However, there are other identities for those whose internal sense lies somewhere between or outside a neat fit into either gender-binary category. Fluidity and fluctuation in gender identity is also recognised, with categories such as ‘non-binary’, ‘gender-fluid’, ‘genderqueer’, ‘pangender’ and ‘genderfuck’ all recorded by clinicians at the UK's Gender Identity Development Service (GIDS) for under-18-year-olds.^22^ The social networking site Tumblr presently describes over 100 different genders.^23^ Without a strong male or female identification, ‘agender’ becomes itself another gender identity.

Some consider gender identity to be fixed and absolute, with some neuroscientists asserting that it develops *in utero* in the second-trimester brain.^24,25^ However, there is little to no convincing evidence to support fundamental differences between the brains of females and males.^26^ If one's ‘internal sense of being a man or a woman’ no longer refers to a ‘man’ or ‘woman’ as defined by biological sex^27^ then the definition of gender identity risks becoming circular.

Within current debates, if gender identity becomes uncoupled from both biological sex and gendered socialisation (Box 1), it develops an intangible soul-like quality or ‘essence’. As a pure subjective experience, it may be overwhelming and powerful but is also unverifiable and unfalsifiable. If this identity is held to be a person's innermost core concept of self, then questioning the very existence of gender identity becomes equated with questioning that person's entire sense of being, and consequently risks being considered a threat to the right to exist, or even as a threat to kill. Behaviours such as ‘misgendering’ or ‘dead-naming’ are understood by proponents of gender theory to be destructive, debasing and dehumanising.^28^ This might explain why the prevailing discourse has become as sensitive and at times inflammatory as it has.

Box 1 Sex, gender and gender identity

**Sex**

Humans are sexually dimorphic: there are only two viable gametes and two sexes, whose primary and secondary sexual characteristics determine what role they play in human reproduction. Sex is determined at fertilisation and revealed at birth or, increasingly, *in utero*. The existence of rare and well-described ‘disorders (differences) of sexual differentiation’ does not negate the fact that sex is binary. The term ‘assigned at birth’ suggests a possibly arbitrary allocation by a health professional, rather than the observed product of sexual reproduction.

**Gender**

Gender describes a social system that varies over time and location and involves shaping of a set of behaviours deemed appropriate for one's sex. It operates at an unconscious level via strong social norms, yet is also rigidly enforced by coercive controls and sometimes violence.^18^ The ‘rules’ exist regardless of how individuals feel about them. Gender can thus be perceived as oppressive and potentially painful to all people of both sexes within patriarchal societies, the dominant form of social structure across most, although not all, of the globe. Feminist theory holds that gender operates as a hierarchy, with men occupying the superior position and women the subordinate. As long as this hierarchy exists, all women are harmed to some extent, whether or not they conform to their sex stereotypes.^19^

**Gender identity**

If sex refers to biology, and gender to socialisation and role, then gender identity may be viewed as the psychological aspect. The American Psychological Association defines it as ‘someone's internal sense of being a man or a woman’.^20^ Gender identity is thus distinguished from biological sex and gendered socialisations.^21^

Nonetheless, notions of gender identity are still contested and raise some ethical questions for professionals working at the interface of physical and mental disorder. Most psychiatrists reject Cartesian dualism, whereby the mind is something imprisoned inside the body, or the ‘ghost in the machine’.^29^ How should doctors consider the body? We are born as, and die as, a body; we *are* our bodies. How can someone be born in the wrong body? Many patients bring a ‘wrong’ or ‘wronged’ body to their doctor; these may be traumatised, wounded, diseased or disliked bodies. How should doctors react when someone informs them that, although they inhabit the body of a man, they are in all other respects female? We must deal with all our patients with compassion but also make safe medical decisions when demonstrable material reality is at odds with a patient's subjectivity.

## Children and adolescents

**Fig. 2:**
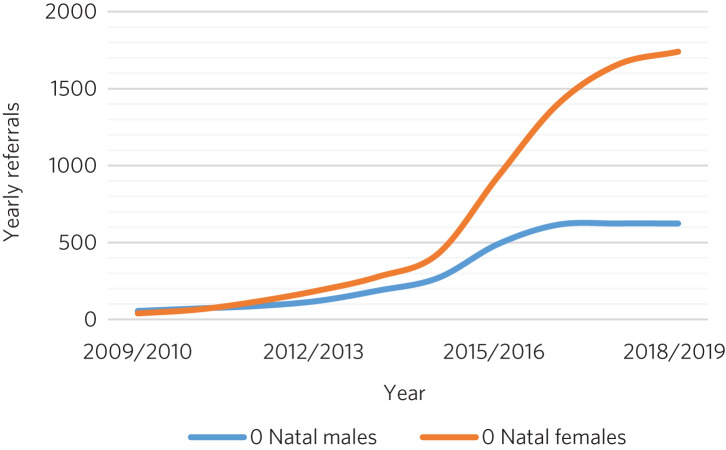
Referral rates to the Gender Identity Development Service at the Tavistock Centre (Tavistock and Portman NHS Trust) in London between 2009 and 2019.^30^

The Gender Identity Development Service (GIDS), Britain's only specialised gender service for children and adolescents and based at the Tavistock Centre, London, has recorded a 25-fold rise in referrals since 2009, most marked in biological girls (‘assigned female at birth’), who make up the majority of referrals presently (Fig. 2).^30^

Despite gender dysphoria no longer falling within the remit of mental illness in ICD-11, there is a substantial body of evidence of increased levels of mental illness among adults, usually attributed to societal responses to gender non-conformity or ‘minority stress’.^31^ De Vries et al measured psychiatric comorbidity among those referred to a child and adolescent gender clinic in The Netherlands and also found increased rates of depression, anxiety and suicidal ideation in this younger group.^32^ However, a potentially worrying picture regarding causes and consequences emerges from more recent research in this young, increasingly natal-female population.

**Fig. 3:**
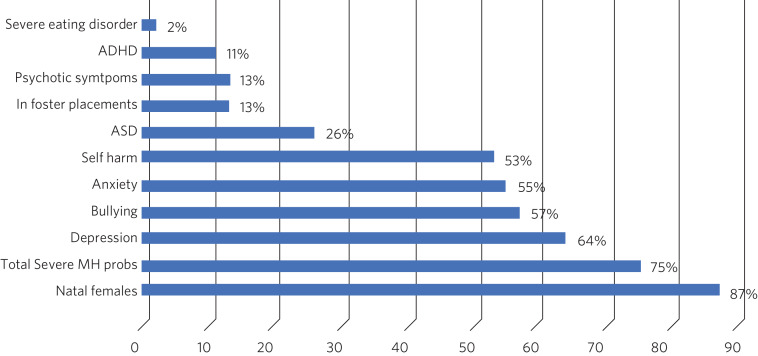
Referrals to an adolescent gender identity clinic in Finland over a 2-year period (from 2011–2013).^33^

Kaltiala-Heino et al examined referrals to an adolescent gender identity clinic in Finland over a 2-year period, finding high rates of mental health problems, social isolation and bullying (Fig. 3).^33^ Most bullying pre-dated the onset of gender dysphoria and was unrelated to gender incongruence.

**Fig. 4:**
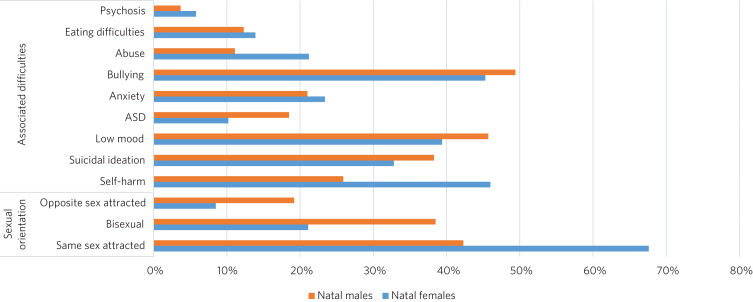
Referrals to the Gender Identity Development Service at the Tavistock Centre (Tavistock and Portman NHS Trust) in London between 1 January 2012 and 31 December 2012.^34^ ASD, autism spectrum disorder.

Similarly, in the UK, Holt et al^34^ found that associated difficulties were common in children and adolescents referred to the GIDS in London (Fig. 4). Same-sex attraction was particularly common among natal females, with only 8.5% of those referred to the GIDS describing themselves as primarily attracted to boys. This raises important questions about current societal acceptance of young lesbians even within youth LGBTQ+ culture. It is possible that at least some gender-non-conforming girls come to believe themselves boys or ‘trans masculine non-binary’ as more acceptable or comfortable explanations for same-sex sexual attraction,^35^ a kind of ‘internalised homophobia’. Autism spectrum disorders are consistently overrepresented in referred children and adolescents.^36^

The RCPsych's position statement acknowledges these elevated rates of mental illness within the transgender population,^1^ but appears to attribute them primarily to hostile external responses to those not adhering to gender norms (or sex-specific stereotypes).^37,38^ A deeper analysis of mental illness and alternative gender identities is not undertaken, and common causal factors and confounders are not explored. This is worrying, as attempts to explore, formulate and treat coexisting mental illness, including that relating to childhood trauma, might then be considered tantamount to ‘conversion therapy’. Although mental illness is overrepresented in the trans population it is important to note that gender non-conformity itself is not a mental illness or disorder. As there is evidence that many psychiatric disorders persist despite positive affirmation and medical transition, it is puzzling why transition would come to be seen as a key goal rather than other outcomes, such as improved quality of life and reduced morbidity. When the phenomena related to identity disorders and the evidence base are uncertain, it might be wiser for the profession to admit the uncertainties. Taking a supportive, exploratory approach with gender-questioning patients should not be considered conversion therapy.

## Suicide, self-harm and current controversies

Transgender support groups have emphasised the risk of suicide. After controlling for coexisting mental health problems, studies show an increased risk of suicidal behaviour and self-harm in the transgender population, although underlying causality has not been convincingly demonstrated.^39^ Then, expressed in the maxim ‘better a live daughter than a dead son’, parents, teachers and doctors are encouraged to affirm unquestioningly the alternative gender for fear of the implied consequences. There is a danger that poor-quality data are being used to support gender affirmation and transition without the strength of evidence that would normally determine pathways of care. One 20-year Swedish longitudinal cohort study showed persisting high levels of psychiatric morbidity, suicidal acts and completed suicide many years after medical transition.^40^ These results are not reassuring and might suggest that more complex intrapsychic conflicts remain, unresolved by living as the opposite sex.

Established risk factors for self-harm and suicidal behaviour appear to be age related (younger trans patients are at higher risk) and include comorbid mental health problems, particularly depression, and a history of sexual abuse.^39^ Thus, all new patients of any age warrant thorough assessment and formulation using a biopsychosocial model; the best evidence-informed interventions should be provided. If this is followed by an individual desisting it should not be considered conversion therapy. That term should perhaps be reserved for coercive treatments.

Best psychiatric practice avoids oversimplification of the causes and treatment of suicidal behaviour and self-harm. Preliminary data from a small ‘before and after’ pilot study of the use of puberty blockers at the Tavistock Centre in selected children found a reduction in body image problems in adolescents following a year of puberty suppression. However, positive effects were offset by increases in self-harm and suicidal thoughts.^41^ Surprisingly, this unpublished study was deemed a success such that prescribing of puberty blockers was introduced as standard practice and commissioned with scaling up of services. There was no development of alternative psychological approaches, nor were randomised controlled comparisons made.

Evidence suggests that almost 100% of children commencing puberty blockade go on to receive cross-sex hormones.^42^ This requires further interrogation to ascertain whether the high figures are due to robust, effective selection and gatekeeping or to a less palatable interpretation that preventing physical and sexual maturation crystallises gender dysphoria as a first step on a cascade of interventions.^43^ The GIDS remains under intense scrutiny regarding research criticisms.^44^ Although in the early 2000s it was criticised for being too conservative and not offering puberty blockers, there appears to have been a volte-face made in response to external pressure,^45^ without the publishing of robust data showing that this intervention is effective and safe.

Puberty blockers are known to affect bone and, possibly, brain development. They put users at risk of developing osteoporosis^46^ and are associated with reductions in expected IQ.^47^ They are described as ‘buying time’ for adolescents to make up their mind about whether to proceed with transition. Long-term effects are not known, but infertility appears inevitable when cross-sex hormones are introduced shortly after puberty blockers.^48^ Loss of sexual maturation will also be associated with lack of adult sexual function, although it is unlikely that a pre-pubertal child can truly understand this side-effect at the time of consent.

Those seeking transition are a vulnerable population who suffer from high levels of suicidality, psychiatric morbidity and associated difficulties. Medical and surgical transition is sought to relieve these psychiatric symptoms. Plausibly, there is an initial reduction in distress following transition, although no controlled trials exist. Therefore, the long-term outcome of medical and surgical transition in terms of mortality and quality of life remains unknown. No long-term comparative studies exist that satisfactorily demonstrate that hormonal and surgical interventions are superior to a biopsychosocial formulation with evidence-based therapy in reducing psychological distress, body dysphoria and underlying mental illness.

## Clinical implications

**Fig. 5:**
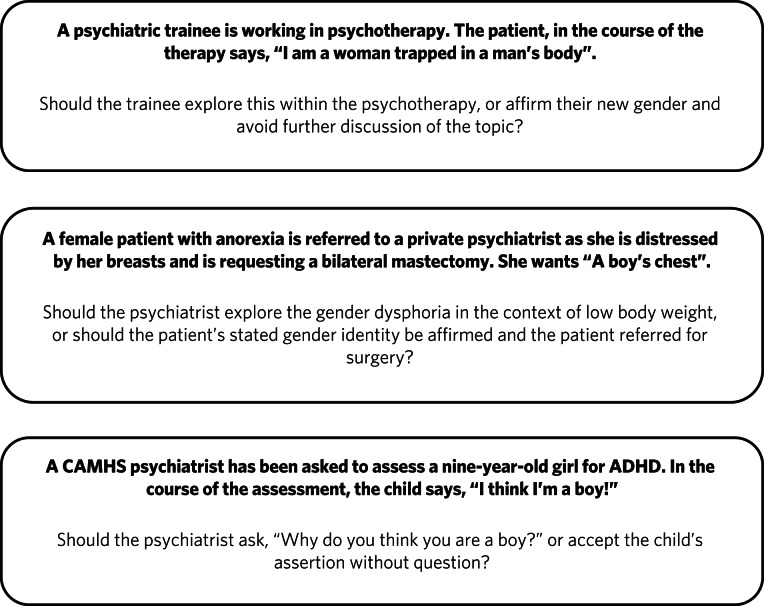
Are these scenarios examples of good clinical practice or conversion therapy?

It is unclear what the role of psychiatry is in the assessment and treatment of gender dysphoria, now that it is no longer considered a diagnosable mental illness, and whether there is still a place for a routine psychosocial assessment. It could be argued that patients should be deterred from gender intervention pathways while comorbid mental illness is treated (Fig. 5). Without long-term follow-up data, it is not possible to identify those who might reconcile with their sex and those who might come to deeply regret their medical and/or surgical transition. Moreover, it is not transparent where ultimate and legal responsibility for decision-making lies – with the patient, parents (if the patient is a child), psychologist, endocrinologist, surgeon or psychiatrist.

Psychiatrists understand that human development is necessary, but not always comfortable. Puberty, although a normal physiological process, is associated with particularly high levels of psychological and bodily discomfort. Psychiatrists’ role is to journey with patients as change is navigated and to provide support through sharing uncertainty and difficult decision-making. But in the current climate, psychiatrists may be unsure whether addressing psychological and social antecedents will lead to accusations of conversion therapy. Attempts to reconcile a sufferer's discomfort with their actual body would be good practice in other conditions involving body image disturbance, such as anorexia nervosa.

The magnitude of any benefits of medical and surgical transition is not clear. Follow-up studies are sparse, and with the new cohort of adolescents, clinicians step even further into the unknown.^49^ These young people are not comparable to adult, mainly male-to-female, research participants on whom existing empirical clinical guidelines were based. Doctors are now questioning the wisdom of gender-affirmation treatment of children and young people, citing poor diagnostic certainty and low-quality evidence.^50^ A recent review of evidence for the use of gender-affirming hormones for children and adolescents states that these drugs ‘can cause substantial harms, including death’ and concludes ‘the current evidence base does not support informed decision making and safe practice’.^51^

**Fig. 6:**
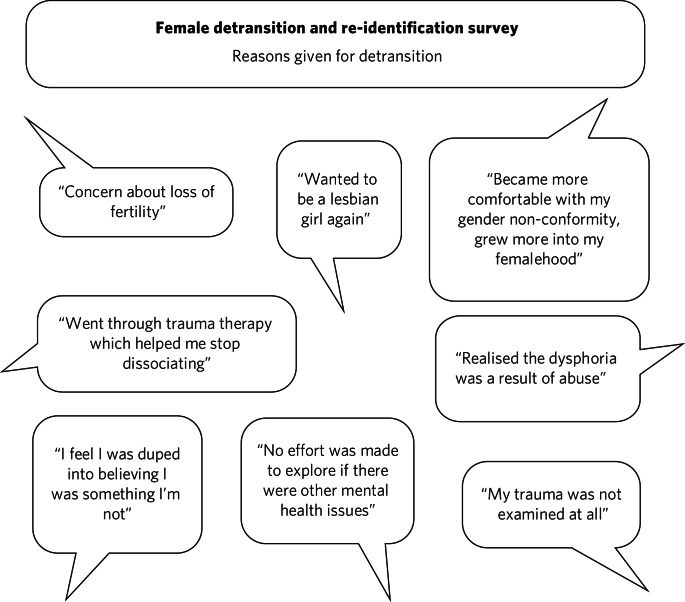
Reasons given for detransitioning in a female detransition and re-identification survey run between 16 and 30 August 2016 and shared through online social networking sites.^52^

Among a plethora of online videos by teenagers proudly displaying their mastectomy scars a worrying increase in detransitioner testimonies can now be found^52^ (Fig. 6). These are mainly young women who have rejected their trans identities and are reconciling with their birth sex.

## Feminist concerns

In theory, universal human rights should not pit disadvantaged groups against one another, but in practice, disputes occur. Women's rights activists point to persistent global inequalities, sex discrimination and violence against women and girls. They are concerned that ignoring sex as a reality risks no longer being able to name, measure and ameliorate sex-based harms. Endorsing old sex and gender stereotypes in an attempt to validate young patients may inadvertently shore up outdated notions of how men and women should look and behave. There is no reason to believe that women have an innate love of pink and wearing high heels and find map-reading difficult, any more than men have a natural leaning towards blue and playing football and make excellent leaders.

Inherent in the notion of ‘gender identity’ is that there already exists a specific subjective experience of being a man or a woman. However, there cannot be a significant intrinsic experiential difference between male and female human beings when we cannot know what those differences are. One cannot possibly know how it feels to be anything other than oneself. Medicine may be in danger of reinforcing social norms and reifying a concept that is impossible to define over and above material biological reality. At present, many health, social, educational and legal policies are being adapted to give gender primacy over sex.^53–57^

## Conclusions

Language that confuses or conflates sex and gender identity, while appearing inclusive, might have the unintended consequence of closing down the means to understand complexity and respond appropriately to patients’ emotional and material reality. The medical profession must be compassionate, accept differences and fight for those who are marginalised and discriminated against.

However, viewing transgender as a fixed or stable entity, rather than a state of mind with multiple causative factors, closes down opportunities for doctors and patients to explore the meaning of any discomfort. Being gender non-conforming, or wishing to opt out of gender altogether, is not only *not* indicative of mental disorder – it is, in many ways, an entirely rational response to present capitalist reliance on rigid gender norms and roles. However, when multiple medical interventions are required on an otherwise healthy body or doctors are expected to deny the concept of sex or the sexed body, the situation becomes less coherent. The notion of conversion therapy for those seeing themselves as transgender relies on another binary – that of ‘cisgender’ and ‘transgender’ – being set, closed, biologically anchored categories without overlap, rather than a more plausible hypothesis that one's gender identity is flexible, informed by one's culture, personality, personal preferences and social milieu.

The push for early bodily modification and hormones by some transgender patients is a cause for concern. New services, modelled on commissioning guidance from NHS England for adults of 17 years and above, will allow for self-referral, preclude psychological formulation or therapeutic intervention as standard practice, and recommend hormonal intervention after two appointments.^58^ This will further scale up hormonal and surgical interventions in young patients, who will miss out on pubertal development and necessary mental health treatment in their quest for interventions that may harm and that they may later regret.

In the rapidly moving and politicised debate, psychiatrists look to the RCPsych for guidance. Those providing and interpreting the scanty evidence from published research need to be independent and impartial, using best-quality measures rather than ideology. It is confusing to liken open-minded working with young patients as they figure out who they are to conversion therapy. Holding an empathic neutral middle ground, which might or might not include medical transition, should not be equated with this. Psychiatrists need to feel empowered to explore the meaning of identity with their patients, treat coexisting mental illness and employ a trauma-informed model of care when appropriate.

The General Medical Council's Good Medical Practice demands of clinicians compassion, shared decision-making and safeguarding of young people's open futures.^59^ The counterargument to unquestioning gender affirmation is that the process of medical transition may itself prove to be another form of conversion therapy, creating a new cohort of life-long patients dependent on medical services and turning at least some lesbian and gay young people into simulacra of straight members of the opposite sex. Psychiatry sits on this knife-edge: running the risk of being accused of transphobia or, alternatively, remaining silent throughout this uncontrolled experiment. Respectful debate, careful research and measurement of outcomes are always required.

## About the authors

**Lucy Griffin**, MBBS, BSc, MRCPsych, is a consultant psychiatrist at The Priory Hospital Bristol, UK. **Katie Clyde**, MBChB, MRCPsych, DGM, is a consultant psychiatrist with Southern Health NHS Foundation Trust, Hampshire, UK. **Richard Byng**, MB BChir, MRCGP, MPH, PhD, is a general practitioner and Professor of Primary Care Research at the University of Plymouth, UK. **Susan Bewley**, MD, FRCOG, MA, is Professor (Emeritus) of Obstetrics and Women's Health, Department of Women & Children's Health, King's College London, UK.
